# Overweight and Obesity Coexist with Thinness among Lao's Urban Area Adolescents

**DOI:** 10.1155/2020/5610834

**Published:** 2020-08-14

**Authors:** Katiya Ivanovitch, Sonemany Keolangsy, Nontiya Homkham

**Affiliations:** Faculty of Public Health, Thammasat University (Rangsit Campus), Klong Luang District, Patumthani 12120, Thailand

## Abstract

**Introduction:**

In recent decades, the developing countries of Southeast Asia, including the Lao People's Democratic Republic (Lao PDR), have experienced a rapid growth of their urban population. Partly as a result of that, issues of undernutrition and overnutrition became a significant public health problem.

**Objective:**

To examine the prevalence of overweight and obesity and their related factors, among the school-attending adolescents in the Lao capital of Vientiane.

**Methods:**

A cross-sectional data on 300 adolescents aged 15–19 were collected during the months of March, April, and May 2018 by means of a self-administrated questionnaire. Anthropometric measurements were used to obtain data on height and weight. Pearson's chi-squared test, Fisher exact tests, and univariable and multivariable logistic regressions were applied in the course of the statistical analysis.

**Results:**

The study found a high prevalence of overweight/obesity (23.3%) and thinness (10.3%). Poor eating habits were noted in 67.0% of adolescents, even though 78.0% of them had a good knowledge of nutrition. Factors significantly associated with the overweight/obesity were low physical activities (aOR = 18.3; 95% CI: 5.51–60.66) and adolescents living with their guardians (aOR = 0.25; 95% CI: 0.08–0.79). Results also indicated that, in 47.3% of the cases, teachers, acting as a source of health and nutrition information, can prevent the risk of adolescents' overweight/obesity (aOR = 2.05, 95% CI = 1.11–3.80) but not their thinness (aOR = 0.4, 95% CI = 0.17–0.88).

**Conclusions:**

Laotian adolescents are facing the spectrum of malnutrition in urban areas. To improve adolescents' nutritional status, there is a need for a collaborative approach of public health agencies that would address the issues of an effective food and nutrition policy. The school curricula should also include programs on nutrition and physical education.

## 1. Introduction

In its new global assessment of child malnutrition, the UNICEF is warning about high rates of childhood obesity in a rising number of low- and middle-income countries. In East and Southeast Asia, increasing urbanization and socioeconomic development have led to changes in eating habits and physical activities associated with rising obesity and high undernutrition in adults and children [1]. The World Health Organization (WHO) also indicates that overweight and obesity among adolescents aged 5–19 had risen from just 4% in 1975 to more than 18% in 2016 [2]. That is an ominous trend because adolescent obesity has negative consequences for physical and mental health [3], and it is strongly linked with obesity during adulthood [4]. The double burden of malnutrition has emerged as a worldwide concern where undernutrition and overnutrition coexist in same populations and households. In the Asia-Pacific region, this has become a significant public health issue, mirroring the WHO 2017 report that a one-third of low- and middle-income countries were facing a double burden of malnutrition [5]. That emerging public health problem leads to increased mortality, a higher risk of chronic diseases, and rising healthcare costs [6].

The Lao PDR, a Southeast Asian nation with 6.8 million inhabitants, has experienced a strong economic growth that made it possible to move from a low-income to a low middle-income country [7]. In 2011, 33% of Laotians lived in urban areas, compared with an average of 49% in Southeast Asian developing countries. The Sisattanak district of Vientaine, where this study was conducted, is part of the Sam Sang pilot project called “The building of provinces as strategic units, districts as the comprehensively strengthened units, and villages as development units.” As part of its development projects, the government laid out the National Nutrition Policy (NNP), with the focus on reducing child malnutrition and integrating the NNP in “The 8^th^ Five Years National Economic Strategic Development Plan” for the period of 2016 to 2020. The program is falling behind in its efforts to “end hunger, achieve food security, improve nutrition, and promote sustainable agriculture [8].” Adolescents are particularly vulnerable to problems of poor diets and inactive lifestyles. They have high nutritional requirements to support their growth and development. It is, therefore, crucially important to monitor their nutritional status before their health is seriously compromised. The problem, however, is that there is a very limited data available on the nutritional status of Lao's adolescents. The Lao Social Indicator Survey (LSIS) 2011-12 is the only major source of the country's nutrition-related data with nutrition information of mothers and children under the age of 5 [9]. According to the LSIS 2011-12, 44% of children under five years of age are stunted and 27% are underweight. These findings provide a clear picture of the severity of adolescents' nutritional problem in Laos [9]. This study is focusing on that important public health issue in an effort to assess the prevalence of overweight, obesity, and thinness among urban area adolescents in Vientiane.

## 2. Materials and Methods

### 2.1. Study Design and Sample

**Figure 1:**
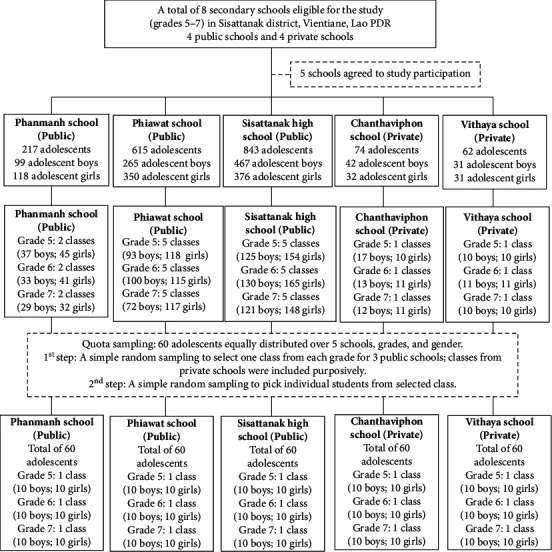
Study's sampling process.

We conducted a quantitative cross-sectional study covering the months of March, April, and May of 2018 among adolescents aged 15–19 years attending public and private high schools in the Sisattanak district of Vientiane. Excluded from the study were (1) students or parents/guardians who refused interviews or anthropometric measurements, (2) students with mental or physical limitations that would inhibit completing the interview and assessments, and (3) students absent from the class at the time of the survey. Schools included into the study were private and public schools that provide grades 5–7 study programs under the authority of the Ministry of Education. Originally, a total of 4 public and 4 private secondary schools were included, with grade 5–7 programs attended by 2,114 students. However, in the end, only 3 public and 2 private schools agreed to participate in the study. The minimum number of 250 students necessary for the investigation was increased by 20% to anticipate the dropout rate. The resulting sample size of 300 high school adolescents was divided equally among 5 schools according to grades and gender. A multistage sampling method was performed. In the first stage, a simple random sampling was conducted to select one class from each grade for 3 public schools. For private schools, classes were included purposively since there was only one class in each grade. In the second stage, students from selected classes were picked randomly to obtain 10 boys and 10 girls by using computer-generated random numbers. If there were less than 10 boys and 10 girls, a second class in the same grade and school were randomly selected until the required sample size was obtained (Figure 1). The sampling frame was students' identification number in their respective schools.

### 2.2. Data Collection

Self-administered questionnaires were used to collect information from students and their parents/guardians. The questionnaire was used to obtain background information on students, their anthropometric measurements, nutrition knowledge, nutrition attitude, eating habits, sources of nutrition information, physical activity patterns, and socioeconomic status of their parents/guardians (i.e., income, education, and occupation). Students were given standardized instructions for filling out the questionnaires by the researcher and trained interviewers. Questionnaires were completed in the classroom, and interviewers were present to answer questions, if necessary.

### 2.3. Measures

The questionnaire was validated and pretested prior to data collection. The questionnaire was given for content validation to 3 experts in the area of study. Their corrections and modifications were incorporated in the final form of the questionnaire. The questionnaire was pretested on 10% (30) adolescent boys and girls in a school other than those included in the sample.

#### 2.3.1. Socioeconomic Conditions

Data were collected on the number of siblings, household size, daily allowance, living arrangements, and education, and occupation of parents/guardians.

#### 2.3.2. Anthropometric Measures

Adolescents' weight and height were recorded by trained research assistants following the standard procedures. Height was measured with a wooden stadiometer to the nearest 0.1 cm. Placed on a flat surface, the children stood on the basal part of the device with feet together (without shoes) and standing upright on bare feet, heels together, buttock and back touching the stadiometer and with their eyes in the horizontal plane. Weight was measured with an electronic scale to the nearest 100 g, with students wearing light clothing and without shoes. To minimize human and instrument errors, height and weight were measured and recorded twice by the same enumerator and averaged for reliability and accuracy. The body mass index (BMI) was defined as weight (kg)/height (m^2^) and transformed into BMI-for-age *z*-scores by using Antro Plus software [10]. BMI-for-age *z*-scores were categorized based on the WHO BMI cutoffs for thinness (BAZ < -2 SD), overweight (BAZ >1 SD and ≤2 SD), and obesity (BAZ >2 SD) were also estimated [11]. In logistic regressions, overweight/obese was defined as BAZ >1 SD.

#### 2.3.3. Nutrition Knowledge

Participants' nutrition knowledge was evaluated using interviews and a 15-item questionnaire. A score of 1 was awarded to questions answered correctly, and 0 (zero) was allocated to wrong answers or “do not know” answers. The questions were about the knowledge, concepts, and processes related to nutrition and health, including knowledge of diet and health and diet for disease prevention. The knowledge score ranged from 0 to 15. The Bloom's cutoff point was used to classify the knowledge into three levels: poor for 59% or below (0–8 scores), moderate for 60–80% (9–12 scores), and good for 80–100% (13–15 scores) [12, 13].

#### 2.3.4. Nutrition Attitude

To assess participants' attitudes toward nutrition and disease prevention, questions were asked regarding the importance of eating specific healthy foods. Attitude domain comprised of 15 Likert scale items. Adolescents could indicate their degree of agreement towards the statement given. The Likert scale of five points was used to represent the scores “strongly agree,” “agree,” “uncertain,” “disagree,” and “strongly disagree”. Numerical scores 5, 4, 3, 2, and 1 were assigned to categories “strongly agree,” “agree,” “not sure,” “disagree,” and “strongly disagree,” respectively. For those items which were negatively phrased, scores were recoded as 5, 4, 3, 2, and 1 for categories “strongly disagree,” “disagree,” “uncertain,” “agree,” and “strongly agree.” The nutrition attitude score ranged from 15 to 75 points, classified into 3 categories “poor,” “moderate,” and “good” by using Bloom's cutoff point [12, 13].

#### 2.3.5. Eating Habits

A food frequency questionnaire was administered, and dietary intake was measured by the consumption of certain specific foods, fruit and vegetable, junk food, and fast food. The eating habit domain consisted of 20 items assessed by “6-7 times per day,” “4-5 times per day,” “2-3 times per day,” “everyday,” “5-6 times per week,”, “3-4 times per week,” “1-2 times per week,” “1–3 times per month,” and “never,” scored as 9, 8, 7, 6, 5, 4, 3, 2, and 1. For negative eating habit items, scores were recoded as 9, 8, 7, 6, 5, 4, 3, 2, and 1 for category “never,” “1–3 times per month,” “1-2 times per week,” “3-4 times per week,” “5-6 times per week,” “everyday,” “2-3 times per day,” “4-5 times per day,” and “6-7 times per day.” Eating habits scores ranged from 20 to 180 and were classified into 3 categories “poor,” “moderate,” and “good” by using the Bloom's cutoff point [12,13].

#### 2.3.6. Physical Activity

Data on the level of physical activity were obtained from the adapted measure of the International Physical Activity Questionnaire (IPAQ)—Short Form [14]. The IPAQ short form asks respondents to report frequency and duration of walking and moderate-intensity and vigorous-intensity activity performed for at least 10 minutes per session. The summary indicator was used to categorise population into three levels of physical activity: “low” (physically inactive), “moderate,” and “high” levels of physical activity. These categories were based on standard scoring criteria [15]. A low level of physical activity meets neither “moderate” nor “high” criteria. The moderate level meets any of the following three criteria: (a) 3 days of vigorous activity of at least 20 minutes/day, (b) 5 days of moderate-intensive activity or walking more than 30 minutes/day for more than 10 minutes at a time, or (c) 5 days of any combination of walking and moderate-intensity or vigorous-intensity activities achieving at least 600 MET-minutes/week. A high level meets either of the two criteria: (a) vigorous-intensity activity on more than 3 days/week and accumulating at least 1500 MET-minutes/week or (b) more than 5 days of any combination of walking, and moderate-intensity or vigorous-intensity activities achieving at least 3000 MET-minutes/week.

### 2.4. Statistical Analysis

Descriptive statistics were used to describe the demographic characteristics of the subjects. Pearson's chi-squared and Fisher exact tests were used to explore general characteristics and differences in nutritional status. Univariable and multivariable binary logistic regression models were used to describe the relationship between subjects' characteristics and abnormal nutritional status (i.e., thinness and overweight/obesity). Crude odds ratio (OR) with a corresponding 95% confidence interval (CI) was calculated from univariable analysis. Factors associated (*p* value <0.10) with outcomes in a univariable analysis to be included into multivariable logistic regression. Adjusted odds ratio with corresponding 95% CI was estimated from multivariable analysis. A backward stepwise selection was applied with the level of significance for variables to remain in the final model set at 0.05. For all statistical tests, a *p* value <0.05 was taken as the level of significance. Any missing data were managed by listwise deletion technique.

### 2.5. Ethical Considerations

The study was approved by the National Ethics Committee for Health Research of the Lao PDR (No 040/NECHR). Participation in the study was voluntary, and all information obtained was treated with standard assurances of confidentiality. Adolescents and their parents or guardians signed separate terms of consent forms.

## 3. Results

### 3.1. General Characteristics of Study Participants

Of the 300 sampled adolescents, there were no refusals or study dropouts. Almost half of adolescents were 17 years old (41.3%), with the average age of 16.93 years (SD ± 1.00), and nearly one-half of them (46.0%) were the first-born. Approximately one-half of adolescents (45.3%) had 2 siblings, and the largest proportion of households (45.0%) consisted of 2 and 4 people. Almost one-half of adolescents (48.3%) had an average daily allowance between 10,001 and 20,000 kips (1.2–2.4 USD). About 7.3% of them reported a chronic disease such as anaemia, asthma, hypertension, and allergies. Almost one-third of them were taking supplements (26.3%), with multivitamins (11.7%), calcium (6.7%), and vitamin C (5.3%). More than one-third of adolescents (76.3%) lived with their parents. Fathers' education level was higher than mothers'. More than half of fathers had a college/university degree (55.3%), but only 34.4% of mothers had the same academic credentials. Most of the fathers were employed as government officers (39.7%), and 39.6% of mothers were unemployed housewives. Most of adolescents (78.0%) had a good knowledge of nutrition. However, the good attitude toward nutrition was found in only 6.3% of respondents. In addition, 67% of adolescents had poor eating habits. Only 1.7% of them had good eating habits. Nearly two-thirds (62.7%) of adolescents reported a low level of physical activity. Surprisingly, 14.0% were overweight and 9.3% were obese, while thinness was found in 10.3% of the cases.

The general characteristics of the studied variables were computed to determine distribution differences among thinness, normal weight, overweight, and obesity at the significance level of *p* value <0.05 (Tables 1 and 2). There were no statistically significant nutritional status differences in general characteristics of studied variables, except for physical activity levels and teachers as a source of nutrition information.

### 3.2. Factors Associated with Thinness and Overweight/Obesity

In this study, the univariable analysis (Table 3) showed those who did not receive nutrition information from teacher (Crude Odds Ratio (cOR) = 0.4; 95% CI: 0.18–0.91) in private school (cOR = 2.72; 95% CI: 1.12–6.6) were associated with thinness. Factors associated with adolescents' overweight/obesity were students in grade 7 (cOR = 2.3; 95% CI 1.12–4.57); adolescents living with guardians (cOR = 0.3; 95% CI: 1.10–0.83), students who did not receive nutrition information from teacher (cOR = 2.1; 95% CI: 1.19–3.68), poor eating habits (cOR = 2.0; 95% CI 1.05–3.78); and low physical activities (cOR = 18.8; 95% CI 5.73–61.84).

Results obtained in the final multivariable logistic regression model (Table 4) showed that adolescents who did not receive nutrition information from teachers had 40% lower odds of being thin (95% CI: 0.17–0.87), and students from private schools showed a 2.9 times higher risk of being thin, compared with those who studied in public schools (95% IC: 1.17–7.08).

Adolescents with low level of physical activity had 18.6 times higher risk of being overweight/obese, compared with those who had normal/high physical activity levels (95% CI: 5.51–61.56). Adolescents living with guardians had a low probability of becoming overweight/obese (aOR = 0.3: 95% CI: 0.08–0.79). Adolescents who did not receive nutrition information from teachers had a 2.1 times higher risk of being overweight/obese (95% CI: 1.11–3.80), compared with those who received such information.

## 4. Discussion

This is the first study among urban adolescents in Lao PDR exploring the prevalence of overweight, obesity, and thinness and their association with several modifiable lifestyle and socioeconomic factors. The findings of the study indicate that 23.3% of boys and girls were affected by overweight/obesity, almost a twofold increase from 13% reported by Phouapanya in a 2015 research conducted among high school students in Vientiane (unpublished data). The results of this study also showed a higher prevalence of overweight/obesity when compared with the global statistic (18%) and the neighbouring Vietnam (17.3%) [16, 17]. The WHO also found that most overweight or obese children lived in developing countries and that this problem was increasing faster than in the developed countries [18]. A possible reason for that is a rapid increase of urbanization and associated lifestyle changes with less physical activity and new diets with micronutrient-poor and high-energy convenience foods. That presents a serious public health issue because overweight in adolescence leads to greater risk of an early onset of chronic disorders, such as type 2 diabetes, high blood pressure, metabolic syndrome, and psychological disorders [19].

It was also found that 10.3% of both genders of adolescents were affected by thinness, confirming the evidence that many developing countries in Southeast Asia faced a double burden of malnutrition [20]. Thinness among adolescents is associated with higher risk of anaemia and infectious diseases [21]. Malnutrition of girls of childbearing age presents pregnancy risks, with intrauterine growth retardation, delivery complications, preterm birth, and maternal mortality [22]. The Lao problem of overweight/obesity and thinness is reflecting a global trend where 192 million children worldwide were found in 2016 to be moderately or severely thin, while 124 million were obese [23]. Similar results were observed in other Southeast Asian countries, such as Vietnam, Cambodia and Myanmar, with, respectively, 14.3%, 10.9%, and 12.9% cases of thinness. That was much higher than 7.7% found in Thailand and Malaysia [24]. This double burden of malnutrition has particularly important implications for public policy in countries experiencing rapid urbanizations and attendant changes in nutrition patterns and traditional lifestyles [25]. Our findings support the notion that interventions are needed to address both the rising obesity in urban areas and the lasting problem of undernutrition.

The result of this study did not find a significant association between adolescents' socioeconomic status, illness history, parents/guardians' educational level, and occupation with occurrence of thinness, overweight, and obesity.

Even though the multivariable logistic regression did not find that grades and eating habits were a significant factor determining thinness and overweight/obesity, a univariable analysis had shown that class grades and eating habits were significantly associated with overweight/obesity. In particular, adolescents attending higher class grades were found to have a higher risk of overweight/obesity. That might be ascribed to the possibility that they are able to more readily access food by themselves. The study found that adolescents' overall nutrition knowledge was good, with a moderate level of nutrition attitude and poor eating habits. But, our findings indicate no significant association between nutrition knowledge and nutrition attitude with good eating behaviour. Adolescents knew that fried foods, fizzy sweetened drinks, potato chips, and processed foods were unhealthy and bad sources of energy and nutrients. In spite of that the negative attitude toward unhealthy foods, adolescents still continued to consume those kinds of foods at least once a week. One can conclude from those findings that the study participants were knowledgeable about healthy and unhealthy foods and drinks, but they were unable to apply that knowledge to modify their daily dietary choices. These results are consistent with those found by Doak [26]. Eating habits are the key factor influencing the risk of overweight and obesity in this study. More than half (67%) of the study subjects showed poor eating habits reflected in their consumption of high energy-dense foods, too many fried foods and soft drinks with excessive fat, salt, and sugar. The prominent examples of such foods are sticky rice, pizza, fried chicken, and instant noodles. This confirms an old observation that adolescents are the main fast food consumers, a habit that may lead to vitamin deficiency, eating disorders, and diet-related pathologies [27]. In our survey, two-thirds of respondents ate fast food at least once a week, and only 30% reported eating fresh fruits and vegetables every day. Similar surveys found that only one-third of school-attending adolescents were eating vegetables in five Southeast Asian countries like India, Indonesia, Thailand, and Myanmar [28]. Our research indicates that there is a statistically significant relationship between eating habits and nutritional status. Adolescents with poor eating habits had a 2.0 times higher risk of overweight/obesity than those presenting moderate to good eating habits. That showed that adolescents' improper eating behaviour led to excessive energy intakes and to subsequent problems of overweight and obesity [29]. Adolescents' nutrition status, eating habits, and lifestyle practices, including physical activity, are also greatly influenced by their sources of nutrition information.

Multivariable analysis showed the crucial role played by the type of school attended by adolescents. Those findings indicated that adolescents who enrolled in private school were 2.9 times more likely to be thin, with a lower risk of overweight/obesity as compared with adolescents attending public (government) schools. Our results contradict studies finding higher prevalence of thinness among public schools and higher cases of overweight in private schools [30–32]. In the context of the Lao PDR, there is no difference between environments and study programs in government and private schools. Government and private school canteens are usually outsourced to commercial enterprises, resulting in a lack of effective contribution by the school management in menu planning. The type of school attended served as a proxy for the socioeconomic status of parents or guardians. That was based on the assumption that rich parents were more likely to send their children to private schools. But, our study found that there was no difference in student's daily allowances between government and private schools. This might be due to differences in the feeding habits and student's self-awareness of their personal image. Another important finding of our study is that adolescents who received nutrition information from teachers were better able to avoid becoming overweight/obese than those who did not. That is consistent with several studies showing that educators had an important role in supporting students' healthy food choices and in helping them to attain a healthy weight status [33–35].

Schools have been identified as powerful platforms for supporting students' physical and psychological well-being [36]. Educators, therefore, have an active role to play in intervention strategies promoting healthy nutrition and lifestyles [37].

The level of physical activity is one of the key determinants of nutritional status. Inadequate physical activity can be an important contributing factor to the development of overweight and obesity. Adolescents with sedentary lifestyles or low physical activity levels were 18.6 times more likely to be overweight/obese than those who had moderate and high levels of physical activities. These results are in line with research reported by Desalew A. et al., who found that children in Ethiopia without a regular physical exercise were 3.8 times more likely to develop a significant risk of overweight/obesity [38]. Spending time sitting for over 3 hours on weekends (watching television, playing games, etc.) led to lower metabolic rates and frequent snacking [39, 40]. Our study shows that only 13% of adolescents engaged in regular physical activity. That finding is consistent with research indicating that in 146 countries 77.6% of boys and 84.7% of girls led physically inactive lifestyles [41].

Physically active lifestyles during adolescence can produce long-term health benefits and prevent many noncommunicable diseases, such as obesity, cardiovascular disease, cancer, and diabetes. It is, therefore, recommended that adolescents should do at least 60 minutes of moderate-to vigorous‐intensity physical activity daily. Anything less than that is considered insufficient [42].

Living arrangements also have a substantial impact on adolescents' nutritional status. Those living with their parents had higher risks of overweight/obesity than those who did not live with their parents. Parents are one of the most important environmental factors that influence the eating behavior and adolescents' risk of overweight/obesity [43]. That was particularly the case of almost 40 percent of Lao adolescents whose mothers were housewives. As reported in the study by Alison [44], mothers feeding behaviour puts emphasis on pleasing the child and showing that she was a successful, effective, and cherished parent. A study conducted by Burton also found that caregivers had the influence on adolescents' nutrition status [45]. Parents are determining the supply of food, and they influence their children's eating behaviour. Both factors were shown to play a crucial role in prevention and treatment of obesity [34].

### 4.1. Study Strengths

This is the first analysis to examine nutritional status and associated factors of urban area adolescents attending private and public schools in Lao PDR. In that sense, the study fills an important gap because most nutrition information available so far referred to mothers and their children under the age of 5 [46, 47]. If left unattended, problems of adolescents' nutritional status found by this study could have negative public health consequences, with adverse effects on the country's workforce and economic development. Early detection of weight problems would allow decision makers to implement interventions to reduce associated morbidity and mortality. The body mass index (BMI) limitations to distinguish between fat mass and muscle mass [48] have been mitigated by anthropometric measurements, which is a noninvasive and inexpensive way to measure the nutritional status of children and adolescents.

### 4.2. Study Limitations

This was a cross-sectional survey where any associations cannot be interpreted as causal. Reverse causation is also possible. Our analysis includes only school-attending adolescents. That means that our findings cannot be representative of the entire Lao adolescent population. But, the inclusion of subgroups, such as out-of-school adolescents, was not possible because of the lack of data about this largely understudied population [49]. In addition, we calculated our estimates from self-reported data, which are known to exhibit reporting flaws [50, 51]. In addition, dietary intakes were not reported in the study; their inclusion would be appropriate in further studies.

## 5. Conclusions

Our study shows that 23.3% of Lao PDR adolescents attending private and public schools in one of the districts of the capital city of Vientiane were overweight/obese, while 10.3% of the same population subgroup was affected by thinness. It was also found that their low physical activity levels raised the odds of overweight/obesity. Adolescents living with guardians were less likely to become overweight/obese. School teachers as a source of nutrition information were shown as a protective factor against overweight/obesity, but that did not prevent thinness. The cases of thinness were more present among adolescents attending private schools.

This study has important implications for dealing with the nutritional status of urban Lao adolescents. Special attention has to be paid to public health problems caused by declining levels of physical activity and increasing consumption of fast foods with a high content of fats, sugar, and sodium. Parents, guardians, and teachers have a crucial role to play in promoting healthy nutrition and lifestyles within this population subgroup. Teachers are shown to be an important source of nutrition information, which means that instruction about nutrition and active lifestyles should become part of the regular school curricula. There is also a need to move toward more inclusive concepts, such as nutrition literacy among adolescents, because nutrition knowledge and attitude toward nutrition are not always correlated with healthy eating behaviours. Future public health intervention programs would do well to adopt a multisectoral approach in dealing with challenging problems of overweight, obesity, and undernutrition.

## Figures and Tables

**Table 1:** The prevalence of thinness, normal weight, overweight, and obesity according to its determinants.

General characteristics	All (*n* = 300)		Thinness (*n* = 31)		Normal (*n* = 199)		Overweight (*n* = 42)		Obese (*n* = 28)		*p*-value
	*N*	%	*n*	%	*n*	%	*N*	%	*N*	%	
Gender											0.821^a^
Boy	150	50.0	17	11.3	96	64.0	23	15.3	14	9.3	
Girl	150	50.0	14	9.3	103	68.7	19	12.7	14	9.3	

Age (years)											0.473^b^
15	27	9.0	3	11.1	18	66.7	5	18.5	1	3.7	
16	65	21.7	3	4.6	49	75.4	8	12.3	5	7.7	
17	124	41.3	13	10.5	82	66.1	18	14.5	11	8.9	
18	70	23.3	10	14.3	44	62.9	8	14.4	8	14.4	
19	14	4.7	2	14.3	6	42.9	3	21.4	3	21.4	

Grade											0.054^a^
Grade 5	100	33.3	9	9.0	75	75.0	10	10.0	6	6.0	
Grade 6	100	33.3	9	8.0	66	66.0	19	19.0	7	7.0	
Grade 7	100	33.3	9	14.0	58	58.0	13	13.0	15	15.0	

Birth order											0.941^a^
1^st^ birth	138	46.0	14	10.1	90	65.2	21	15.2	13	9.4	
2^nd^ birth	100	33.3	9	9.0	67	67.0	13	13.0	11	11.0	
>2^nd^ birth	62	20.7	8	12.9	42	67.7	8	12.9	4	6.5	

Number of siblings											0.429^a^
1-2	166	55.3	16	9.6	108	65.1	28	16.9	14	8.4	
>2	134	44.7	15	11.2	91	67.9	14	10.4	14	10.4	

Household members											0.111^a^
2–4	135	45.0	17	12.6	85	63.0	24	17.8	9	6.7	
>4	165	55.0	14	8.5	114	69.1	18	10.9	19	11.5	

Daily allowance (kip) (1$ = 8,00 kip)											0.881^a^
5,000–10,000	88	29.3	11	12.5	60	68.2	9	10.2	8	9.1	
10,001–20,000	145	48.3	13	9.0	94	64.8	24	16.6	14	9.7	
>20,000	67	22.3	7	10.4	45	67.2	9	13.4	6	9.0	

History of chronic diseases											0.390^b^
No	278	92.7	23	9.7	184	66.2	41	14.7	26	9.4	
Yes	22	7.3	4	18.2	15	68.2	1	4.5	2	9.1

Taking supplements											0.412^a^
No	221	73.7	24	10.9	142	64.3	31	14.0	24	10.9	
Yes	79	26.3	7	8.9	57	72.2	11	13.9	4	5.1	

Living arrangement											0.316^b^
Parents	230	76.7	27	10.5	164	63.8	36	15.7	24	10.4	
Single parental	27	9.0	4	14.8	17	63.0	4	14.8	2	7.4	
Nonparental guardians	43	14.3	4	9.3	35	81.4	2	4.7	2	4.7	

Mother's education (*n* = 250)											0.098^a^
Below college level	164	65.6	21	12.8	99	60.4	24	14.6	20	12.2	
College/university	86	34.4	5	5.8	60	69.8	16	18.6	5	5.8	

Father's education (*n*-237)											0.175^a^
Below college level	106	44.7	10	9.4	67	63.2	13	12.3	16	15.1	
College/university	131	55.3	14	10.7	85	64.9	23	17.6	9	6.9	

Guardian's education (*n* = 43)											0.253^b^
Below college level	16	37.2	3	18.8	13	81.3	0	0.0	0	0.0	
College/university	27	62.8	1	3.7	22	81.5	2	7.4	2	7.4	

Mother's occupation (*n* = 250)											0.800^a^
Government	47	18.8	4	8.5	28	59.6	11	23.4	4	8.5	
Nongovernment	104	41.6	10	9.6	67	64.4	15	14.4	12	11.5	
Unemployed	99	39.6	12	12.1	64	64.6	14	14.1	9	9.1	

Father's occupation (*n* = 237)											0.805^b^
Government	94	39.7	10	10.6	67	71.3	11	11.7	6	6.4	
Nongovernment	138	58.2	15	10.9	88	63.8	20	14.5	15	10.9	
Unemployed	5	2.1	0	0.0	3	60.0	1	20..0	1	20.0	

Guardian's occupation (*n* = 43)											0.117^b^
Government	18	41.8	0	0.0	16	88.9	1	5.6	1	5.6	
Nongovernment	22	51.2	4	18.2	17	77.3	0	0.0	1	4.5	
Unemployed	3	7.0	0	0.0	2	66.7	1	33.3	0	0.0	

School type											0.115^a^
Government	180	60.0	24	13.3	111	61.7	27	15.0	18	10.0	
Private	120	40.0	7	5.8	15	12.5	10	8.3	88	73.3	

Nutrition knowledge											0.770^a^
Fair	66	22.0	9	13.6	43	65.2	8	12.1	6	9.1	
Good	234	78.0	22	9.4	156	66.7	34	14.5	22	9.4	

Nutrition attitude											0.285^a^
Poor	62	20.7	10	16.1	39	62.9	5	8.1	8	12.9	
Fair	219	73.0	19	8.7	148	67.6	35	16.0	17	7.8	
Good	19	6.3	2	10.5	12	63.2	2	10.5	3	15.8	

Eating habit											0.081^a^
Poor	201	67.0	17	8.5	129	64.2	22	10.9	33	16.4	
Fair	99	33.0	14	14.1	70	7.7	9	9.1	6	6.1	

Physical activity											<0.001^*∗*^^a^
Low	188	62.7	13	6.9	108	57.4	40	21.3	27	14.4	
Moderate	73	24.3	16	21.9	56	76.7	1	1.4	0	0.0	
High	39	13.0	2	5.1	35	89.7	1	2.6	1	2.6	

^a^ *p* value by Pearson chi-squared test; ^b^*p* value by Fisher exact test; ^*∗*^*p* value is significant at <0.05.

**Table 2:** The prevalence of thinness, normal weight, overweight, and obesity according to adolescents' sources of nutrition information.

Source of information	All (*n* = 300)		Thinness (*n* = 31)		Normal (*n* = 199)		Overweight (*n* = 42)		Obese (*n* = 28)		*p*-value^a^
	*n*	%	*n*	%	*n*	%	*n*	%	*N*	%	
Social media											0.936
No	35	11.7	4	11.4	22	62.9	6	17.1	3	8.6	
Yes	265	88.3	27	10.2	177	66.8	36	13.6	25	9.4	

Television											0.452
No	105	35.0	11	10.5	75	71.4	12	11.4	7	6.7	
Yes	195	65.0	20	10.3	124	63.6	30	15.4	21	10.8	

Teacher											0.012^*∗*^
No	158	52.7	21	13.3	92	58.2	25	15.8	20	12.7	
Yes	142	47.3	10	7	107	75.4	17	12.0	8	5.6	

Friends											0.545
No	160	53.3	20	12.5	105	65.6	20	12.5	15	9.4	
Yes	140	46.7	11	7.9	94	67.1	22	15.7	13	9.3	

Family members											0.287
No	164	54.7	16	9.8	111	67.7	26	15.9	11	6.7	
Yes	136	45.3	15	11	88	64.7	16	11.8	17	12.5	

Newspaper or magazine											0.838
No	210	70.0	22	10.5	142	68	28	13.3	18	8.6	
Yes	90	30.0	9	10	57	63.3	14	15.6	10	11.1	

Textbook											0.287
No	224	74.7	24	10.7	142	63.4	35	83.3	23	10.3	
Yes	76	25.3	7	9.2	57	75	7	9.2	5	6.6	

Radio											0.356
No	266	88.7	30	11.3	176	66.2	37	13.9	23	8.6	
Yes	34	11.3	1	2.9	23	67.6	5	14.7	5	14.7	

Health program activities in school											0.923
No	231	77.0	24	10.4	152	65.8	34	14.7	21	9.1	
Yes	69	23.0	7	10.1	47	68.1	8	11.6	7	10.1	

Health program activities in community											0.373
No	266	88.7	30	11.3	173	65	37	13.9	26	9.8	
Yes	34	11.3	1	2.9	26	76.5	5	14.7	2	5.9	

^a^ *p* value by Pearson chi-squared test; ^*∗*^*p* value is significant at <0.05.

**Table 3:** A univariable analysis of factors associated with the nutritional status of adolescents.

Variables	Univariable logistic regression			
	Thinness		Overweight/Obesity	
	cOR (95% CI)	*p* value	cOR (95% CI)	*p* value
Gender (girl§)				0.507
Boy	0.8 (0.36–164)	0.495	1.2 (0.70–2.08)	

Grade (Grade 5§)				0.088
Grade 6	1.0 (0.36–2.71)	0.984	1.9 (0.91–3.74)	
Grade 7	0.5 (0.20–1.22)	0.130	2.3 (1.12–4.57)	0.023^*∗*^

Age (year) (15–17§)				0.307
18-19	0.5 (0.24–1.17)	0.117	1.4 (0.75–2.48)	

Birth order (>2^nd^ birth)				0.477
1^st^ and 2^nd^ birth	1.3 (0.54–3.12)	0.556	1.3 (0.64–2.63)	

Daily allowance (kip) (>20000 kip§) (1$ = 8000 kip)				0.838
≤20000	1.0 (0.40–2.46)	0.997	1.1 (0.55–2.07)	

History of chronic diseases (no§)				0.355
Yes	0.6 (0.17–1.78)	0.319	6 (0.51–1.96)	

Food supplements (no§)				0.243
Yes	1.4 (0.56–3.37)	0.485	0.7 (0.36–1.30)	

Living arrangement (parents§)				0.022^*∗*^
Guardians	1.4 (0.47–4.38)	0.520	0.3 (1.10–0.83)	

Father's education (college/university§)				0.646
Below college level	1.1 (0.46–2.64)	0.825	1.2 (0.63–2.09)	

Mother's education (college/university§)				0.443
Below college level	0.4 (0.41–1.10)	0.074	1.3 (0.69–2.33)	

Guardians' education (college/university§)				NA
Below college level	0.2 (0.02–2.10)	0.178	NA	

Father's occupation (government§)				0.158
Nongovernment	1.6 (0.66–3.74)	0.302	1.6 (0.83–3.09)	

Mother's occupation (government§)				0.469
Nongovernment	1.0 (0.28–3.31)	0.945	0.8 (0.35–1.63)	
Unemployed	0.8 (0.23–2.57)	0.661	0.7 (0.31–1.48)	0.320

Guardians' occupation (government§)				0.871
Nongovernment	NA	NA	0.8 (0.11–6.67)	

Number of siblings (1-2§)				0.407
>2	0.9 (0.42–1.92)	0.782	0.8 (0.46–1.38)	

Household members (2–4§)				0.521
>4	1.6 (0.76–3.49)	0.209	0.8 (0.48–1.45)	

Sources of nutrition information				0.061
Textbook (yes§)				
No	0.7 (0.30–1.78)	0.485	1.9 (0.94–3.88)	

Newspaper or magazine (yes§)				0.377
No	1.0 (0.44–2.35)	0.964	0.8 (0.43–1.38)	

Television (yes§)				0.113
No	1.1 (0.50–2.42)	0.813	0.6 (0.34–1.12)	

Radio (yes§)				0.550
No	0.3 (0.03–1.96)	0.189	0.8 (0.35–1.74)	

Family members (yes§)				0.673
No	1.2 (0.55–2.52)	0.665	0.9 (0.52–1.54)	

Teacher (yes§)				0.010^*∗*^
No	0.4 (0.18–0.91)	0.029^*∗*^	2.1 (1.19–3.68)	

Friends (yes§)				0.691
No	0.6 (0.28–1.35)	0.225	0.9 (0.52–1.54)	

Social media (yes§)				0.685
No	0.8 (0.27–2.62)	0.763	1.2 (0.52–2.72)	

Health program activities in community (yes§)				0.503
No	0.2 (0.03–1.70)	0.147	1.4 (0.56–3.27)	

Health program activities in school (yes§)				0.708
No	0.9 (0.38–2.32)	0.899	1.1 (0.59–2.19)	

School type (government§)				0.216
Private	2.72 (1.12–6.6)	0.027^*∗*^	07 (0.39–1.23)	

Nutrition knowledge (good§)				0.777
Poor and fair	0.7 (0.29–1.57)	0.360	0.9 (0.46–1.78)	

Nutrition attitude (fair and good§)				0.852
Poor	0.5 (0.22–1.17)	0.114	0.9 (0.47–1.88)	

Eating habit (fair and high§) (adjusted)				0.035^*∗*^
Poor	1.5 (0.71–3.26)	0.285	2.0 (1.05–3.78)	

Physical activity level (moderate and high§)				<0.001^*∗*^
Low	1.6 (0.76–3.54)	0.204	18.8 (5.73–61.84)	

^§^Reference group (normal weight), ^*∗*^*p* value is significant at <0.05; cOR: crude odds ratio.

**Table 4:** A multivariable logistic regression analysis of factors associated with adolescents' nutritional status.

Variables	Multivariable logistic regression			
	Thinness		Overweight/Obesity	
	aOR (95% CI)	*p* value	aOR (95% CI)	*p* value
Living arrangement (parents§)				0.017^*∗*^
Guardians	1.4 (0.43–4.28)	0.608	0.3 (0.08–0.79)	

Teacher (yes§)				0.023^*∗*^
No	0.4 (0.17–0.87)	0.022^*∗*^	2.1 (1.11–3.80)	

Physical activity level (moderate and high§)				<0.001^*∗*^
Low	1.8 (0.83–3.99)	0.139	18.6 (5.51–61.56)	

School type (government§)				0.422
Private	2.9 (1.17–7.08)	0.022^*∗*^	0.8 (0.40–1.47)	

^§^Reference group (normal weight), ^*∗*^*p* value is significant at <0.05, aOR: adjusted odds ratio.

## Data Availability

The data analysed for this manuscript are available from the corresponding author and can be made accessible upon reasonable request.

## References

[1] Haddad L., Cameron L., Barnett I. (2015). The double burden of malnutrition in SE Asia and the Pacific: priorities, policies and politics. *Health Policy and Planning*.

[2] World Health Organization (2020). *Obesity and Overweight*.

[3] Obesity Collaborators G. B. D., Afshin A., Forouzanfar M. H. (2017). Health effects of overweight and obesity in 195 countries over 25 years. *The New England Journal of Medicine*.

[4] The N. S., Suchindran C., North K. E., Popkin B. M., Gordon-Larsen P. (2010). Association of adolescent obesity with risk of severe obesity in adulthood. *JAMA*.

[5] World Health Organization (2020). *More than One in Three Low-and Middle-Income Countries Face Both Extremes of Malnutrition*.

[6] Kolčić I. (2012). Double burden of malnutrition: a silent driver of double burden of disease in low-and middle-income countries. *Journal of Global Health*.

[7] World Bank (2019). *World Bank Country and Lending Groups*.

[8] Von Grebmer K. (2020). *Global Hunger Index*.

[9] Ministry of Health and Lao Statistics Bureau (2012). *Lao Social Indicator Survey 2011-12*.

[10] World Health Organization (2009). *WHO AnthroPlus for Personal Computers Manual: Software for Assessing Growth of the World’s Children and Adolescents*.

[11] World Health Organization (2007). *BMI-for-Age (5–19 Years)*.

[12] Abeje T., Negera E., Kebede E. (2016). Performance of general health workers in leprosy control activities at public health facilities in Amhara and Oromia States, Ethiopia. *BMC Health Services Research*.

[13] Abdullahi A., Hassan A., Kadarman N., Saleh A., Shu’aibu Y. u. B., Lua P. L. (2016). Food safety knowledge, attitude, and practice toward compliance with abattoir laws among the abattoir workers in Malaysia. *International Journal of General Medicine*.

[14] Craig C. L., Marshall A. L., Sjöström M. (2003). International physical activity questionnaire: 12-country reliability and validity. *Medicine & Science in Sports & Exercise*.

[15] IPAQ Research Committee (2005). *Guidelines for Data Processing and Analysis of the International Physical Activity Questionnaire (IPAQ)–Short and Long Forms Contents*.

[16] World Health Organization (2016). *Prevalence of Overweight Among Children and Adolescents*.

[17] Nguyen P. V., Hong T. K., Hoang T., Nguyen D. T., Robert A. R. (2013). High prevalence of overweight among adolescents in Ho Chi Minh City, Vietnam. *BMC Public Health*.

[18] World Health Organization (2019). *Commission on Ending Childhood Obesity*.

[19] Reilly J. J., Kelly J. (2011). Long-term impact of overweight and obesity in childhood and adolescence on morbidity and premature mortality in adulthood: systematic review. *International Journal of Obesity*.

[20] (2017). *Strategic Action Plan to Reduce the Double Burden of Malnutrition in the South-East Asia Region 2016–2025*.

[21] Dobner J., Kaser S. (2018). Body mass index and the risk of infection-from underweight to obesity. *Clinical Microbiology and Infection*.

[22] Han Z., Mulla S., Beyene J., Liao G., McDonald S. D. (2011). Maternal underweight and the risk of preterm birth and low birth weight: a systematic review and meta-analyses. *International Journal of Epidemiology*.

[23] Abarca-Gómez L., Abdeen Z. A., Hamid Z. A. (2017). Worldwide trends in body-mass index, underweight, overweight, and obesity from 1975 to 2016: a pooled analysis of 2416 population-based measurement studies in 128·9 million children, adolescents, and adults. *The Lancet*.

[24] World Health Organization (2014). *Prevalence of Thinness Among Children and Adolescents, BMI <−2 Standard Deviation below the Median, Crude Estimates by Country, Among Children 5–19 Years*.

[25] Zhang Y.-X., Wang Z.-X., Zhao J.-S., Chu Z.-H. (2016). Prevalence of overweight and obesity among children and adolescents in Shandong, China: urban-rural disparity. *Journal of Tropical Pediatrics*.

[26] Doak C. M., Visscher T. L., Renders C. M., Seidell J. C. (2006). The prevention of overweight and obesity in children and adolescents: a review of interventions and programmes. *Obesity Reviews: An official Journal of the International Association for the Study of Obesity*.

[27] Washi S. A., Ageib M. B. (2010). Poor diet quality and food habits are related to impaired nutritional status in 13-to 18-year-old adolescents in Jeddah. *Nutrition Research*.

[28] Peltzer K., Pengpid S. (2012). Fruits and vegetables consumption and associated factors among in-school adolescents in five Southeast Asian countries. *International Journal of Environmental Research and Public Health*.

[29] Sahoo K., Sahoo B., Choudhury A. K., Sofi N. Y., Kumar R., Bhadoria A. S. (2015). Childhood obesity: causes and consequences. *Journal of Family Medicine and Primary Care*.

[30] Shakya T., Jha C. B., Koirala S. (2016). Comparison of stunting and thinness among early adolescents from government and private schools of Dharan, Nepal. *International Journal of Health Sciences and Research*.

[31] Bhattarai S., Bhusal C. K. (2019). Prevalence and associated factors of malnutrition among school going adolescents of Dang district, Nepal. *AIMS Public Health*.

[32] Pal A., Pari A. K., Sinha A., Dhara P. C. (2017). Prevalence of undernutrition and associated factors: a cross-sectional study among rural adolescents in West Bengal, India. *International Journal of Pediatrics and Adolescent Medicine*.

[33] Pike K. M., Dunne P. E. (2015). The rise of eating disorders in Asia: a review. *Journal of Eating Disorders*.

[34] Cunha D. B., Souza B. d. S. N. d., Pereira R. A., Sichieri R. (2013). Effectiveness of a randomized school-based intervention involving families and teachers to prevent excessive weight gain among adolescents in Brazil. *PLoS One*.

[35] Price C., Cohen D., Pribis P., Cerami J. (2017). Nutrition education and body mass index in grades K-12: a systematic review. *Journal of School Health*.

[36] Langford R., Bonell C. P., Jones H. E. (2014). The WHO Health Promoting School framework for improving the health and well-being of students and their academic achievement. *Cochrane Database of Systematic Reviews*.

[37] Bruss M., Dannison L., Morris J. R. (2010). Teachers as partners in the prevention of childhood obesity. *International Journal of Education Policy and Leadership*.

[38] Desalew A., Mandesh A., Semahegn A. (2017). Childhood overweight, obesity and associated factors among primary school children in dire dawa, Eastern Ethiopia; a cross-sectional study. *BMC Obesity*.

[39] Adesina A. F., Peterside O., Anochie I., Akani N. A. (2012). Weight status of adolescents in secondary schools in port Harcourt using body mass index (BMI). *Italian Journal of Pediatrics*.

[40] Prentice-Dunn H., Prentice-Dunn S. (2012). Physical activity, sedentary behavior, and childhood obesity: a review of cross-sectional studies. *Psychology, Health & Medicine*.

[41] Guthold R., Stevens G. A., Riley L. M., Bull F. C. (2020). Global trends in insufficient physical activity among adolescents: a pooled analysis of 298 population-based surveys with 1·6 million participants. *The Lancet Child & Adolescent Health*.

[42] World Health Organization (2018). *Physical Activity*.

[43] Jiang M.-H., Yang Y., Guo X.-F., Sun Y.-X. (2013). Association between child and adolescent obesity and parental weight status: a cross-sectional study from rural North China. *Journal of International Medical Research*.

[44] Kalinowski A., Krause K., Berdejo C., Harrell K., Rosenblum K., Lumeng J. C. (2012). Beliefs about the role of parenting in feeding and childhood obesity among mothers of lower socioeconomic status. *Journal of Nutrition Education and Behavior*.

[45] Burton E. T., Wilder T., Beech B. M., Bruce M. A. (2017). Caregiver feeding practices and weight status among African American adolescents: the Jackson heart KIDS pilot study. *Eating Behaviors*.

[46] Annim S. K., Imai K. S. (2014). Nutritional status of children, food consumption diversity and ethnicity in Lao PDR. *The School of Economics Discussion Paper Series 1404, Economics*.

[47] Lao Statistics Bureau (2018). *Lao Social Indicator Survey II 2017, Survey Findings Report*.

[48] Shah N. R., Braverman E. R. (2012). Measuring adiposity in patients: the utility of body mass index (BMI), percent body fat, and leptin. *PLoS One*.

[49] Guthold R., Moller A.-B., Azzopardi P. (2019). The global action for measurement of adolescent health (GAMA) initiative-rethinking adolescent metrics. *Journal of Adolescent Health*.

[50] Hidding L. M., Chinapaw M. J. M., Van Poppel M. N. M., Mokkink L. B., Altenburg T. M. (2018). An updated systematic review of childhood physical activity questionnaires. *Sports Medicine*.

[51] Gemming L., Jiang Y., Swinburn B., Utter J., Mhurchu C. N. (2014). Under-reporting remains a key limitation of self-reported dietary intake: an analysis of the 2008/09 New Zealand adult nutrition survey. *European Journal of Clinical Nutrition*.

